# Knowledge, attitudes and practices survey on organ donation among a selected adult population of Pakistan

**DOI:** 10.1186/1472-6939-10-5

**Published:** 2009-06-17

**Authors:** Taimur Saleem, Sidra Ishaque, Nida Habib, Syedda Saadia Hussain, Areeba Jawed, Aamir Ali Khan, Muhammad Imran Ahmad, Mian Omer Iftikhar, Hamza Pervez Mughal, Imtiaz Jehan

**Affiliations:** 1 Medical College, The Aga Khan University, Karachi, Pakistan; 2 Department of Community Health Sciences, The Aga Khan University, Karachi, Pakistan

## Abstract

**Background:**

To determine the knowledge, attitudes and practices regarding organ donation in a selected adult population in Pakistan.

**Methods:**

Convenience sampling was used to generate a sample of 440; 408 interviews were successfully completed and used for analysis. Data collection was carried out via a face to face interview based on a pre-tested questionnaire in selected public areas of Karachi, Pakistan. Data was analyzed using SPSS v.15 and associations were tested using the Pearson's Chi square test. Multiple logistic regression was used to find independent predictors of knowledge status and motivation of organ donation.

**Results:**

Knowledge about organ donation was significantly associated with education (p = 0.000) and socioeconomic status (p = 0.038). 70/198 (35.3%) people expressed a high motivation to donate. Allowance of organ donation in religion was significantly associated with the motivation to donate (p = 0.000). Multiple logistic regression analysis revealed that higher level of education and higher socioeconomic status were significant (p < 0.05) independent predictors of knowledge status of organ donation. For motivation, multiple logistic regression revealed that higher socioeconomic status, adequate knowledge score and belief that organ donation is allowed in religion were significant (p < 0.05) independent predictors. Television emerged as the major source of information. Only 3.5% had themselves donated an organ; with only one person being an actual kidney donor.

**Conclusion:**

Better knowledge may ultimately translate into the act of donation. Effective measures should be taken to educate people with relevant information with the involvement of media, doctors and religious scholars.

## Background

Organ transplantation saves thousands of lives worldwide. According to WHO, kidney transplants are carried out in 91 countries. Around 66,000 kidney donations, 21,000 liver donations and 6000 heart donations were transplanted globally in 2005 [1]. Organs for donation are procured from both living donors as well as cadavers. In South-East Asia, and Pakistan, however, almost all organ donations come from living donors [2].

Pakistan is a developing Muslim country of more than 160 million people [3]. According to the estimates of a prominent kidney transplants centre of Pakistan, Sindh Institute of Urology and Transplantation (SIUT), approximately 15,000 patients in Pakistan suffer from kidney failure every year. The only treatment options available for these patients are either dialysis or kidney transplantation [4]. As of 2007, there are 12 transplantation centers in Pakistan with five being in the public sector and seven in the private sector. Approximately 400 renal transplants are done every year despite the increasing number of patients with end stage renal disease (ESRD); the donors being living. According to available statistics, only seven cadaveric kidneys from abroad have been harvested for transplantation so far and only one from a local cadaver [2].

It is s dismal fact that there is no liver transplantation centre in the country [5] despite the high estimated prevalence of Hepatitis B and Hepatitis C in our population; being 3–4% and 6% respectively [6,7]. Data about the transplantation of other organs in Pakistan are unfortunately not available. An absence of an organized and well established national registry is a major hurdle in this regard.

Organ transplantation has recently drawn attention as a bioethical issue for robust debate in Pakistan. Emerging concerns intertwined with it include the burgeoning trend of transplantation, lack of legislation to govern it and exploitation of human rights. These efforts led to the promulgation of an Ordinance in 2007 to regulate the transplantation of human organs and tissues [8-10]. This ordinance mentions living donors of at least eighteen years of age. Any close relative can be a donor according to it but must donate voluntarily and without duress or coercion. This law also allows that cadavers can be used as a source of transferable organs in Pakistan [2]. In this Ordinance, "brain dead" means "irreversible loss of brain and brain stem functions simultaneously" while a person will be deemed to be medically and legally dead when there is "an absence of natural respiratory and cardiac functions and attempt at resuscitation are not successful in restoring those functions; or an irreversible and permanent cessation of all brain-stem functions and future attempt of resuscitation or continued supportive maintenance would not be successful in restoring such natural functions" [11]. This Ordinance also makes provisions for the establishment of a regulatory Monitoring Authority for organ transplantation in the country [11]. However, this Ordinance has not yet addressed the establishment or the development of an organ distribution system like UNOS in USA.

The law is important to protect the impoverished sections of the society from exploitation. A survey of kidney vendors done in Punjab, Pakistan showed that 34% were living below the poverty line. Most of these kidney vendors were illiterate; 69% were bonded laborers. Their monthly income was US$ 15.4 ± 8.9. Ninety three percent of these individuals had vended their kidneys for the purpose of debt repayment [12]. Another study reported the various aspects of 104 kidney vendors in Pakistan; 67% were bonded laborers earning < $ 50 per month. Hepatitis B and C positivity was seen in 5.7% and 27% respectively [13]. According to estimates, paid donation makes up 50% of all transplants in Pakistan [14]. Wider public awareness of this Ordinance is important for its reinforcement and implementation. Organ trade is an important emerging issue that should be tackled with appropriate legislation. According to World Health Organization (WHO), organ trafficking may be accounting for up to 5–10% of the kidney transplants performed annually [15].

Overall, globally the prevalence of knowledge for organ donation ranges from 60% to 85% using different knowledge variables [16]. This trend has been reported to vary with the development status of the country. Motivation to donate has been shown to have an association with knowledge and awareness of organ donation [16]. Most of the research evidence on this subject is from the more developed countries. In a study from USA that included 278 respondents, 69.1% knew that blood-type made a difference in donation (p = 0.000), 61.6% knew that transplant survival rates were high (p = 0.000), and 75.9% knew that transplants could come from living donors (p = 0.000) [17]. Another study done in European Union determined that more educated, younger age, and expressing some sort of political affiliation determined willingness to donate one's own organs and consent to the donation of those of a relative [18]. From the developing world, a study conducted in Filipinos using qualitative theme analysis identified major themes related to organ donation as: awareness of organ donation, family beliefs, religion/spirituality, attitude/emotions, personal experience with organ donation, health profession, and cultural issues [19].

There is dearth of information on this subject in Pakistan. Only one study [16] was conducted to gain insight into KAP regarding organ donation among the patients coming to the outpatient units of a tertiary care hospital in Karachi. However, this study focused on the outpatient population coming to clinics and not the general population. This study reported that 59.9% of the people surveyed were willing to donate their organs.

Therefore, the aim of our study was to fill the gaps regarding public awareness of organ donation in Pakistan. Also, we wished to determine factors that motivate or dissuade Pakistani individuals from organ donation. This information would be helpful for tailoring more precisely targeted programs and campaigns in the future.

## Methods

### Study Design and Study Setting

A cross sectional survey was conducted at five conveniently selected market places of Karachi including Tariq Road, Saddar, Bahadarabad, Clifton and market areas in the vicinity of Stadium Road. Karachi, the largest city of Pakistan, is a nucleus of various commercial activities with a number of prominent market places. These market places are visited by people of diverse cultural, ethnic, linguistic and socioeconomic backgrounds.

### Sample Size and Sampling Method

A sample size of 385 was calculated assuming a prevalence of 50% for knowledge, attitudes and practices of organ donation, a 95% confidence interval and a sample error of 5%. This was adjusted for 15% non-response rate; bringing the total sample size to 440. Convenience sampling was used to draw the sample for this survey. All consenting individuals, visiting the aforementioned market areas of Karachi between 3 pm to 7 pm and falling in the age bracket of completed 18 to completed 60 years of age were interviewed. Socio-demographic data from the non-respondents including their gender, age and education was also collected.

### Method of Data Collection

Information was collected using face to face interviews based on a structured, pre-tested questionnaire. Pre testing was done on adults falling in the same age brackets, in a similar setting, to screen for potential problems in the questionnaire. The interviewers discussed the questionnaire thoroughly among themselves before data collection to decrease interviewer bias. With the exception of a few open ended questions, the interview was based on prompted questions.

### Questionnaire

The questionnaire was divided into three sections with the first two sections comprising the socio demographic information while section three assessed KAP of organ donation. [see Additional file 1] The individuals were divided into high, middle and low socio-economic classes on the basis of eight variables. This was done because a single variable can't adequately reflect the socioeconomic status of an individual. The variables used included the place of residence of the respondent, presence of basic amenities at home such as clean potable water, electricity, natural gas, 3 square meals a day and adequate sanitation system, ownership of the house, level of education, employment status, cumulative monthly household income, personal means of transport and number of dependant members of the family. Organ donation was defined as "the removal of the tisssues or organs of the human body from a cadaver or from a living donor, for the purpose of transplanting or grafting them into other persons" [20]. Donation from deceased individuals has been defined as per Transplantation of Human Organs and Tissues Ordinance 2007 [11].

Effective legislation was taken to mean a legislation which achieves what it sets out to achieve, meets its designated objectives, and delivers the requisite outcomes.

### Knowledge, Attitude and Practice Variables

Knowledge of the respondents was assessed through questions regarding meanings of the terms "organ donation", awareness of donation by living people as well as cadavers, risks involved in organ donation, and the sources of information for their knowledge. Attitudes of the respondents regarding organ donation was determined through questions regarding opinions on issues such as the willingness to donate organs in the future, influence of religion on attitude towards organ donation, allowance for incentive based organ donation, and factors influencing choice of recipient for future donation. Practices were admeasured by enquiring about actual donation of any organ and any untoward effects observed by individuals in the process that they attribute to organ donation.

### Statistical Analysis

Descriptive statistics, frequency, means (SD) etc were estimated as appropriate. Crude associations were assessed using Odds ratio, Pearson Chi -square test and t-test. All P values were considered significant at < 0.05. Variables with a significant p-value were further evaluated using multiple logistic regression analysis to determine their adjusted association with awareness of organ donation, and motivation to donate. All odds ratios were reported with a 95% confidence interval. Tables and figures were used for an all-inclusive viewing of results.

### Ethical Considerations

The study was given ethical approval by Ethical Review Committee as well as the Department of Community Health Sciences at AKUH. All ethical requirements including confidentiality of responses and informed consent were stringently ensured throughout the project.

## Results

A total of 495 individuals were approached for participation in our survey, 55 (11.1%) declined to participate in the study. Table 1 provides details of the socio-demographic characteristics of our non-respondent population.

**Table 1:** Socio-demographic characteristics of Non-respondents

Socio demographic Variables	Frequency (n = 55)	%
**Mean Age in years**		
1. Males	34.8 ± 5.9 (SD)	NA
2. Females	28.4 ± 4.9 (SD)	NA

**Gender**		
1. Male	24	43.
2. Female	31	56.4

**Education**		
1. Till class 12	29	52.7
2. Graduate/Post graduate/Diploma	19	34.5
3. Informal Education	4	7.3
4. Illiterate/Can only read and write	3	5.5
**Religion**		
1. Islam	50	91
2. Christianity	3	5.5
3. Hinduism	2	3.5

Of the 440 individuals who gave consent to participate in the survey, 408 completed the full interview. The sociodemographic characteristics of our study population (n = 408) are described in table 2. Most of the participants were Muslims (97%).

**Table 2:** Socio Demographic characteristics of Study Population

**Socio-demographic Variables**	**Frequency **(n = 408)	**%**
**Gender**		
Males	261	64
Females	147	36

**Mean Age in years**		
Males/Females	32/33.5	NA/NA

**Marital status**		
1. Currently single	175	42.9
2. Married	233	57.1

**Income(in rupees)**		
1. < 5000	60	14.7
2. > Rs. 5, 000 – 20,000	126	30.8
3. >20,000–50,000	101	24.8
4. >50,000–80,000	69	16.7
5. > Rs. 80,000	52	13

**Occupation**		
1. Currently Employed	232	56.9
2. Currently Unemployed	176	43.1

**Level of education**		
1. Till class 12	174	42.6
2. Graduate/Postgraduate/Diploma	213	52.2
3. Informal Education	6	1.5
3. Illiterate/Can only read and write name	15	3.7

**Religion**		
Islam	396	97
Christianity	8	2
Hinduism	4	1

**Socio economic Status**		
1. High	73	17.9
2. Middle	237	58.1
3. Low	98	24

Participants who hadn't heard of the term "Organ Donation" were not asked to answer other questions of the Organ Donation section. They were included among respondents who were not adequately knowledgeable about organ donation.

Two hundred and forty five people (60%) in this survey achieved an adequate knowledge score for Organ Donation while 163 (40%) had inadequate knowledge. These cumulative scores were based on a set of questions for each organ donation; people achieving ≥ 50% score were regarded as being adequately knowledgeable while those achieving less than 50% scores were regarded as being inadequately knowledgeable. Education and socioeconomic status (SES) were both found to have a significant association with knowledge scores of organ donation (Education: p value: .000, SES: p value: 0.038). Table 3 shows the proportion of respondents with adequate and inadequate knowledge in relation to different socio-demographic variables.

**Table 3:** Knowledge Score of Organ Donation By Socio-demographic Variables

**Socio-demographic Variables**	**Knowledge Status of Organ Donation**				
	Adequate Knowledge Score		Inadequate Knowledge Score		
	Frequency	%	Frequency	%	P-
	(n = 245)		(n = 163)		value

**Age (in years)**					
- 18–28	116	47.4	71	43.6	
- 29–39	64	26.1	47	28.9	0.676
- 40–50	39	15.9	31	19	
- 51–60	26	10.6	14	8.5	

**Gender**					
- Male	156	63.7	105	64.4	0.878
- Female	89	36.3	58	35.6	

**Education**					
- Illiterate/Can only read and write name	5	2	10	6.1	
- Up to class 12	85	34.7	89	54.7	
- Graduation/Post Graduate/Diploma	152	62	61	37.4	0.000
- Informal education	3	1.2	3	1.8	

**Occupation**					
Currently employed	141	57.6	91	55.8	0.510
Currently unemployed	104	42.4	72	44.2	

**Socioeconomic Status**					
High	50	20.4	23	14.1	
Middle	146	59.6	91	55.8	0.038
Low	49	20	49	30.1	
**Religion**					
Islam	238	97.1	158	97	
Christianity	5	2	3	1.8	0.91
Hinduism	2	0.9	2	1.2	

Eighty one (50.1%) people knew that organs for donation can come from cadavers while 36.5% knew that organs for donation can come from living persons. However, only 23% of the people knew that organs for donation can come from both living persons as well as cadavers. Our study showed that 66.2% people knew that kidneys can be donated, followed by 51.5% who knew that blood can be donated and 46.4% who knew eyes can be donated. Only 26.2% of the people knew that kidneys, blood, heart, eyes, liver, skin, bone marrow and lungs can all be donated.

**Figure 1:**
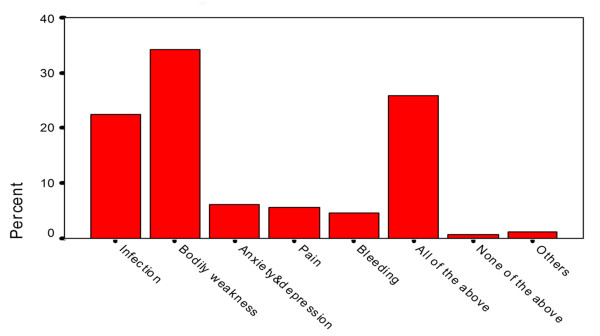
**Risks of Organ Donation**.

In response to the query, "who would you like to donate your organs to?", people reported that they would donate their organs to a family member (51.1%), non-smoker (46.8%), non-drinker (55%), younger age person (less than 30 years old, 40.2%), person belonging to their own religion (32.1%) and a person who is mentally sound (43.9%) and without any physical disabilities (36.8%). With regards to knowledge regarding the various risks associated with organ donation, 55.8% people were aware that organ donation is associated with some risk for the donor. However, 28.7% said that organ donation involves no risks. Among the risks, bodily weakness (34.1%) and infection (22.3%) were the two leading causes chosen by the respondents to be associated with organ donation as shown in figure 1. About 25% knew that organ donation could be associated with all of bodily weakness, infection, bleeding, pain, anxiety and depression.

The attitudes among our study population towards various aspects of organ donation are illustrated in table 4. With regards to allowance of organ donation in religion, there was an almost tri-modal response distribution with about one third responded "yes", about one third "no" and almost one third "don't know". In response to a separate question directed towards identifying the most important factors that people were going to consider before donating an organ to anyone, the two most important factors that emerged were (1)- religion of the recipient: 94 (29.6%) and (2)- the assurance that their organs would be treated respectfully: 87 (27.4%). One hundred and eighty (56.8%) people opined that organ donation should be promoted. Of the 97 people who felt that organ donation should not be promoted, religious beliefs were cited as the leading cause (45.4%).

**Table 4:** Attitude variables regarding Organ Donation

**Attitudinal variables**	**Frequency**	**%**
**Allowance of organ donation in religion **(n = 317)		
-Yes	104	32.8
-No	100	31.5
-Don't Know	113	35.6

**Would like to donate to **(n = 317)		
-Family	162	51.1
- Stranger/Anyone	139	43.8
- Friend/Colleague	16	5.1

**Most important factor for donation **(n = 317)		
- Religion of recipient	94	29.6
- Relationship to recipient	29	9.1
- Age of recipient	18	5.7
- Health status of recipient	40	12.6
- Drug Abuse by recipient	13	4.1
-Assurance of respectful treatment of donated organs	87	27.4
-None of these factors	36	11.3

**Promotion of organ Donation **(n = 317)		
-Needed	180	56.8
-No need	97	30.6
-Don't Know	40	12.6

**Reasons Why Organ Donation Shouldn't Be Promoted **(n = 97)		
-Fear that organs could be wasted/mistreated	22	22.6
-Religious beliefs	44	45.4
-Can lead to organ trade/violation of rights	12	12.4
-Other reasons	19	19.6
(Postoperative pain, family refusal, detest bodily mutilation, and consider it harmful for the donor)		

For knowledge status of respondents, the following variables were subjected to the multiple regression analysis: 'education', and 'socioeconomic status'. Table 5 shows that higher education level and higher socioeconomic status emerged as significant independent predictors of knowledge status of respondents.

**Table 5:** Multiple logistic regression analysis showing independent predictors of knowledge score of organ donation

Socio-demographic Variables	Adequacy of	Adjusted	95%
	Knowledge	OR *	CI *
	n	(%)	
**Education (p = 0.000)**			
Illiterate or Can read and write name only	5 (2)	1	
Up to grade 12	85 (34.7)	1.9	0.6 – 5.8
Graduation/Post graduation/Diploma	152 (62)	5	1.6 – 15.2
Informal Education	3 (1.2)	2	0.3 – 13.7

**Socioeconomic Status (p = 0.038)**			
Low	49 (20)	1	
Middle	146 (59.6)	1.6	0.9 – 2.6
High	50 (20.4)	2.2	1.1 – 4.1

* OR = Odds Ratio

** CI = Confidence Interval

For the motivation status of respondents, the following variables were subjected to multiple logistic regression: 'socioeconomic status', 'knowledge score of organ donation' and 'perceived allowance of organ donation in religion'. Table 6 shows that higher socioeconomic status, adequate knowledge scores and perceived allowance of organ donation in religion emerged as significant independent predictors of knowledge status of respondents.

**Table 6:** Multiple logistic regression analysis showing independent predictors of motivation to donate

Variables	Motivation to Donate	Adjusted	95% CI *
	n (%)	OR *	
**Socioeconomic Status (p = 0.004)**			
			
Low	34 (17.2)		1
Middle	119 (60.1)	1.6	0.9 – 2.8
High	45 (22.7)	2.4	1.1 – 4.9

**Knowledge Score for Organ Donation**			
**(p = 0.002)**			
			
Inadequate Knowledge Score	33 (16.7)	1	
Adequate Knowledge Score	165 (83.3)	2.5	1.5 – 4.3

**Perceived allowance of Organ**			
**Donation in Religion (p = 0.000)**			
			
Don't Know	82 (41.4)	1	
No	32 (16.2)	0.2	0.1 – 0.3
Yes	84 (42.4)	1.7	0.9 – 3.2

* OR = Odds Ratio

** CI = Confidence Interval

**Figure 2:**
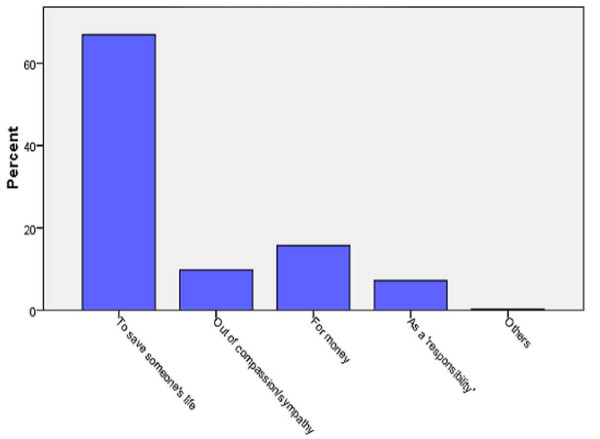
**Reasons for Organ Donation**.

The responses of the respondents with regards to the reasons underlying organ donation are illustrated in figure 2. Almost 60% believed that the basic aim of organ donation is to save someone's life. Some people responded that organ donation can be done out of compassion/sympathy while others cited monetary benefits as the leading motivation behind organ donation. Still some others though that organs are donated as a responsibility.

With regards to the role of the doctor in the process of organ donation process, about 54% of the respondents felt that the doctor should adequately educate the donor as well as the recipients of the risks involved in organ transplantation and then let them make the decision themselves.

With regards to motivation to donate, 120 (37.7%) people said that they would never like to donate any organ while 198 (62.3%) people were motivated to donate. Of the 198 people who were willing to donate, 70 (35.3%) were highly motivated, 36 (18.2%) were moderately motivated and 92 (46.5%) were weakly motivated to donate. Religion, gender, age and marital status didn't have a significant association with the motivation to donate. However, SES was found to have a statistically significant association with the motivation to donate (p = 0.004). Similarly, knowledge scores for organ donation were significantly associated with the motivation to donate an organ (p = 0.002). In addition, the perception about the allowance of organ donation in religion was also significantly associated with the motivation to donate (p = 0.000).

With regards to consent, 76% respondents thought that the donor should be the one who can give consent for a living donation. Thirteen percent respondents thought that the family should give this consent while 5% opined that spouse should give this consent. Three percent of the respondents each thought that friends and doctor should be the one giving the consent.

For donation after death, 52.8% of the people thought that family should have the right to make decision for organ donation while 26.1% people believe that no one has the right to make this decision; only 6% felt that the doctor should be the one deciding this. In the case of unclaimed bodies, a majority (35.2%) felt that the charitable organizations should have the right to decide on this issue while 22.3% felt that no one has the right to make such decisions. Unclaimed bodies were not taken to mean bodies in morgues or bodies found dead on the street (which are not candidates for harvesting organs); rather they were "the case of a dead body lying in a hospital or prison and not claimed by any of the near relatives of the deceased person within forty eight hours from the time of death of the concerned person" [21].

Sixty one percent of the respondents felt that parents or guardians can make decisions on the behalf of mentally retarded persons regarding organ donation. With regards to practices of organ donation, 31% of the people interviewed knew someone who had donated a solid organ; the majority being either family members or friends. In our survey, out of 408 people, 3.5% had themselves donated an organ with only one person having donated a kidney and the remaining ten reported donating blood on one or more occasions. If we simply consider the kidney donation (solid organ donation), the percentage of people who have donated a solid organ falls even further to 0.3%. Television was the leading source of information for most people regarding organ donation as shown in Table 7. Only a minority of the respondents reported doctors as being their source of information.

**Table 7:** Sources of Information (Based on multiple choice questions)

Sources of Information	Organ Donation	
	Frequency (n = 317)	%

**Heard from a doctor**	74	23.3

**Internet**	44	13.8

**TV**	190	59.7

**Radio**	29	9.1

**Newspapers**	142	44.7

**Friends/Colleagues**	51	16

**Others**	5	1.6

## Discussion

We aimed to compare the knowledge, attitudes and practices regarding organ donation in a selected adult population of Karachi, Pakistan. Our analysis of the collected data revealed an interesting set of findings.

Our study showed a slightly lower prevalence of adequate knowledge (60%) regarding organ donation when compared to 65.5% reported by an earlier study in Pakistan [16]. This difference can be explained on the basis of two reasons. Firstly, this could be because of the difference in the study population; ours being the non-patient population encountered in the market places of Karachi while the previous survey was done on the patient population coming to a private tertiary care hospital. Secondly, different knowledge variables have been used in our study as compared to the previous study for the assessment of knowledge status of respondents with regards to organ donation. The associations obtained for organ donation with education and socioeconomic status were also consistent with the previous study. A study done in Lagos, Nigeria also reported that 60% respondents were aware of organ donation in general [22].

Only a minority of the respondents were aware that organs for donation can come from both living persons as well as cadavers. This is significantly different from the previous study [16] where up to 84% people knew that organs could come from cadavers and 71.1% thought that organ donation could be carried out during one's lifetime. This difference can be explained by the reasoning that the patient population in the previous Pakistani study is expected to know more about organ donation. This awareness could possibly have arisen from discourses with doctors or nurses or even fellow patients at the hospital on the subject of organ donation and possible donors. Pamphlets encountered at medical centers could have also enhanced the knowledge of the respondents of the previous study regarding organ donation. Personal experience with organ donation after the death of a family member could also aggrandize the level of awareness of the respondents in the previous study. Out respondents in contrast were encountered on the street and while we didn't enquire about the frequency of their hospital visits, we expect their level of understanding of the process of organ donation to not be equally par with their counterparts who were encountered at the hospital in the previous survey.

In our study, 62% individuals were willing to donate an organ. Fifty one percent respondents mentioned that they would like to donate their organs to family members. These percentages are comparable to data obtained in studies from neighboring countries like China. In a study done in China, 49.8% respondents indicated they would be willing to be living organ donors. Sixty two percent individuals designated relatives as their most probable recipients [23]. A study from Qatar reported that the majority of subjects preferred donating organs to their close relatives and friends [24]. For the results in our survey, we can explain this finding on the basis that in Pakistan, joint family system is generally prevalent with most people living in a closely knit system. Donation of the organ to a family member might be viewed as an "imperative" obligation or it might stem from a feeling of love and compassion for the family member. Moreover, this donation could be done simply because a person has faith and confidence that the organ is being given to a deserving recipient whom he has spent time with and has actually seen suffering from the effects of end organ disease. The person could have deterrence towards donating an organ to a stranger because of the lack of certitude or guarantee that the organ will actually go to the most deserving person. Being biased in donating towards family can therefore be viewed as a natural response of man – a social animal – who functions in a society where the basic unit of architecture and the basic building brick is in fact family.

Our study findings are different from data from other developing countries like Nigeria where only 30% of the respondents expressed a willingness to donate in one survey [22]. In a study from Ohio, over 96% of respondents expressed favorable attitudes toward donation [25].

In contrast to the previous Pakistani study [16] which showed a significant association of the willingness to donate with gender; our results didn't demonstrate any association with gender, age or marital status. A study from Nigeria showed that the willingness to donate an organ was significantly associated with younger age (P = 0.002), but not with gender (P = 0.47) [22].

SES and knowledge score for organ donation was found to be associated with motivation to donate. The perception about the allowance of organ donation in religion was significantly associated with the motivation to donate (p = 0.000). People who believed that religion doesn't allow organ donation showed no motivation to donate in the future. Comparison with the previous study also revealed that the most prevalent reason behind the refusal to donate was a "presumed forbiddance in religion". This could be because of the unawareness of the population regarding religious edicts regarding organ donation. A number of Islamic organizations and institution around the globe have issued fatwas and edicts in favor of organ donation; describing it as "an act of merit" [16,26,27].

Fifty seven percent of respondents were in favor of organ donation and its promotion in the future. This is lower when compared to data from a study done in Brazil which reported that 87% of respondents were in favor of organ donation [28]. We can explain this difference on the basis of the reservation some people might have in view of the recent mushrooming growth of organ trade and trafficking in the country. The negativity projected by the image of organ trade can have a detrimental effect even on the organ donation for altruistic purposes because it weakens the fiber of confidence of the people in the transparency and authenticity of the process.

We asked the respondents why they thought organ donation is done. Apart from answering about saving another human's life, some replied that it is done as a responsibility, others though that it is done for monetary gain while others still thought that it is done out of compassion and sympathy. In principle organ donation driven by altruistic purposes is certainly different from vending a kidney from poverty; it is exactly the kind of understanding we were hoping to gauge through this question. Respondents chose different reasons for organ donation; each individual chose an option nearest to his understanding for the reasons that drive organ donation. Monetary gains were juxtaposed with altruism by respondents. This may also highlight the blurring of perceptual boundaries due to the rampant organ trade in the country; hence the need to improve awareness of organ donation for altruistic purposes in the country can't be over emphasized.

It is a disappointing trend to note that only 23.3% people had heard about organ donation, through a doctor. Comparing our results with the previous study done in Pakistan [16], it is clear that television, print media and doctors fall in the same order of frequency with regards to being sources of information for organ donation. Efforts to judiciously increase the participation of doctors in the process should start at the root level. As a first step, the medical curriculum should increase medical students' awareness of the organ shortage problem and how it can be effectively addressed [29]. A study done in California revealed that speaking to a physician about organ donation positively influenced the likelihood to donate an organ [30]. Although we have no study from Pakistan that assesses the knowledge and attitudes of physicians regarding organ donation, studies from other regions show that over 95% of the physicians who responded to a questionnaire based survey supported organ donation in principle. Physicians responded correctly on average to 68.3 per cent of the questions testing knowledge [31].

It was heartening to see that in our study 88.1% of the people expressed the need for effective legislation to govern organ donation practices. Effective legislation is indeed important to regulate the future practices related to organ transplantation in the country; the lack of which has allowed organ trade to spawn in recent years. An extremely small proportion had actually ever donated a solid organ.

### Strengths and limitations

Our study comes at a point in time when organ donation is an actively debated bioethical and medical issue in Pakistan. Therefore, our research is relevant and timely. Ever since the promulgation of the organ transplantation ordinance last year, the general population has started showing keener interest in the subject with a more receptive attitude towards discussing this issue as was seen by the encouraging response rate in our study. This will create a fertile ground for promoting awareness campaigns in the country.

Through our study and its results, we hope to be in a better position to clarify certain ethical issues regarding organ donation in Pakistan. The awareness regarding organ donation in the country can certainly be improved and this in turn can impact the motivation of the people towards organ donation. We state this because our study and previous studies done in other regions of the world have shown that awareness and motivation go hand in hand. Better awareness of organ donation and its various facets can be expected to improve the motivation to donate. Religion is one vehicle that can be used to motivate people towards organ donation. This survey showed the immense influence religion has in fashioning opinions towards organ donation.

We hope that people will translate these statistics into an aspiration to help others through organ donation. The extremely low level of organ donation seen in our survey should serve as an important revelation that despite the increasing prevalence of end-organ diseases in the country, not many organ donations are being carried out in a legitimate manner. We can also state that perhaps people are not as forthcoming about "backdoor" donations done for monetary gains for fear of being reported to authorities.

Even though 60% of the people interviewed in this survey were adequately knowledgeable about organ donation, the remaining 40% still need to be educated. Secondly, this only represents the knowledge level of an urban Pakistani; Karachi being a major cosmopolitan city and commercial hub of Pakistan. Therefore, its denizens can be expected to be better informed as compared to other areas in the country. The average inhabitant of the rural areas of Pakistan may not be quite as well informed about the growing issues of organ donation. The opinions of the people in this survey can help shape future policies regarding organ donation – their wishes, preferences and reservations can all be actively debated at higher forums before germane policies are engineered. This study can also help create more motivation amongst the people for organ donation; this being one of the major hurdles organ transplantation is facing today.

At the same time, we acknowledge the following limitations of our study. Firstly, we used convenience sampling to draw our sample. Convenience sampling is inferior to probability sampling in its representativeness of the population, and this limits the external validity of the study. Although all efforts were made to include subjects from various areas of the city, there is still a chance that this sampling method may have introduced bias. Some sections of the society may not have been "captured" in our survey, particularly socio-economically deprived areas where we expect to find greater gaps in knowledge and practices. Secondly, the information was acquired via a face-to-face interview which was based on a questionnaire. While this may have led to higher rates of completion of the forms because of interviewer's encouragement for optimum completion, it may also have introduced interviewer's bias in the process of data collection despite all efforts to minimize it. Another limitation was that computation of a knowledge score based on correct answers to a set of questions is somewhat arbitrary, does not incorporate differential weightage that may be placed on different questions and has not been validated. We also devised our own scoring system for categorizing people as belonging to lower, middle and higher socioeconomic classes based on a set of eight socioeconomic variables. Nevertheless, we feel that the scores provide a fairly plausible estimate of the degree of knowledge and the socio economic class of an individual.

Motives for donation may be different for brain dead and living donors. One limitation of our study is that these two types of donors were not differentiated in the questionnaire at a few places. Also, in item 19 of the questionnaire that addresses donation after death, we did not give the option of the deceased giving the consent during his lifetime for donation of organs after death. The results of the respondents to these questions should be interpreted with these limitations in mind.

Respondents reported the donation of blood along with donation of other solid organs with regards to the practices of organ donation. We would like to clarify here that although both are "donatable" tissues, the fundamental distinction between the two was very clear to the respondents as they mentioned to the interviewers during the interview process that blood donation was a "routine thing" for many of them while donation of solid organs like kidneys was not a common incidence. Our results regarding the practices of organ donation, where blood and solid organ donation are mentioned together, should be interpreted with this distinction in mind that the motives behind donating blood and donating a solid organ were different and this distinction was clear to the respondents that blood being a renewable tissue can be donated several times while donating a solid organ has a very permanent connotation attached to it.

We have used a quantitative tool to assess knowledge, attitudes and practices in our survey. This approach may pose some methodological problems in the procurement of all the necessary information for this study. Nevertheless, this study forms an important baseline document for future studies and a qualitative tool can be employed in further studies to gauge requisite information.

## Conclusion

We found that awareness of organ donation is correlated with education and socioeconomic status. Motivation to donate in turn is associated with the awareness of organ donation. Religious beliefs are a major factor deterring many people from expressing a motivation to donate.

Measures should be taken to educate people with relevant information, including the benefits of organ donation and possible risks as well so that people can make informed choices in the future. In the absence of adequate baseline information, it is indeed difficult to comment on whether the general population is already aware of this simple facet. Almost 30% of the people were not aware that organ donation carries any risks. People have a right over their body; they should therefore be fully educated about the future repercussions removing any part of their bodies can have on their health. With full disclosure of such information, they can then make the choice of donating an organ to another human being in the noblest spirit of munificence and benevolence.

Television, newspapers and doctors can be used as efficient sources of information. The communication gap between patients and doctors should be bridged for the generation of a more favorable attitude towards organ donation in the population. Policy makers should also involve religious scholars for the mobilization of a favorable public opinion towards organ donation. In addition, a publicly chartered organization may be established to coordinate live organ donation, including donation by altruistic strangers.

## Conflict of interests

The authors declare that they have no competing interests.

## Authors' contributions

TS and SI gained ethical approval and drafted the questionnaire. They were also involved in study design and conception, data collection, data entry, data analysis, manuscript writing and editing. NH, SSH, AAK, MIA, MOI, HPM and AJ were involved in the data collection, data entry and manuscript writing. IJ was involved in study conception, manuscript writing, revision, editing and overall supervision. All authors have read and approved the final manuscript.

## Pre-publication history

The pre-publication history for this paper can be accessed here:



## Supplementary Material

Additional file 1 **Questionnaire**. The questionnaire used in the survey to gauge knowledge, attitudes and practices of the selected adult population regarding organ donation. Click here for file
