# Supplementary material for: Knowledge, attitudes and practices survey on organ donation among a selected adult population of Pakistan
Source: BMC Med Ethics. 2009 Jun 17;10:5. doi: 10.1186/1472-6939-10-5 (PMC2702378; doi:10.1186/1472-6939-10-5)
Supplement: Additional file 1 — Questionnaire. The questionnaire used in the survey to gauge knowledge, attitudes and practices of the selected adult population regarding organ donation. [file 1472-6939-10-5-S1.doc]

**Knowledge, attitude and practices survey on Organ donation among a selected adult population**

***Instructions to be given to the Respondents by the Interviewers before the start of Interview:***

*This is a* ***questionnaire based interview****, and will take approximately* ***10 minutes*** *to complete. Respondents should choose the option they deem most appropriate for each question. Some questions may require them to choose more than one option.*

**ID number**: _____________________________

**Questionnaire**

**Section 1:**

1. Age (in years): _____________

2. Sex:

a. Male b. Female

3. Occupation:

a. Student b. Housewife

c. Government employee d. Non-government employee

e. Volunteer f. Self employed

g. Retired h. Unemployed

4. Education:

a. Primary (till class 5)

b. Secondary (till class 10)

c. Higher secondary (till class 12 or equivalent)

d. Graduation

e. Post graduation studies

f. Informal education

g. Diploma

h. Can read and write name only

i. Illiterate

5. Marital Status:

a. Single (never married) b. Married

c. Engaged to be married d. Divorced

e. Widowed f. Separated

6. Religion:

a. Islam b. Christianity

c. Hinduism d. Others (specify) ________________

**Section 2:**

7. Cumulative monthly household income:-

a. ≤ Rs. 5, 000 b. > Rs. 5, 000 – 20,000

c. > Rs. 20,000- 50,000 d. > Rs. 50,000 – 80,000

e. > Rs. 80,000 – 100, 000 f. ≥ Rs. 100,000

8. Basic amenities of life (*you may choose more than one option*).

a. Clean potable water b. Electricity

c. Sui gas d. Housing (choose one)

- Own / Rent
- No. of rooms:- ____________________

e. 3 square meals a day f. Adequate sanitation

9. No of dependant family members:

a. ≤ 2 b. 3 c. 4

d. 5 e. 6 f. ≥ 7

10. Means of transport used:

a. Public transport b. Personal bicycle

c. Personal motorbike d. Personal car

**Section 3: Organ Donation (Part A)**

11. Have you ever heard of the term **“Organ Donation”**?

a. Yes b. No c. Don’t know

**Please don’t go further if you answered* ***No / Don’t know*** *to the above question.*

12. Your attitude towards the possibility of your own organs being used for **donation?** (Please rate on a scale of 1-4 where 1 represents lowest level and 4 represents highest level of motivation)

a. Would never consider donating an donate (1)

b. Will think about it (2)

c. Would only like to **donate** under other special circumstances (3)

d. Would definitely want to donate irrespective of circumstances (4)

13. If you picked option **d** to **Q 12**, then please specify the special circumstances?

______________________________________________________________________________

14. Does your religion allow **organ donation**?

a. Yes b. No c. Don’t know

15. Do you believe that there is a danger that **donated** organs could be misused, abused or misappropriated?

a. Never b. Sometimes c. Often

d. Most of the time e. All the time

16. Who would you like to **donate** your organs to? (*Please pick one option from each set*)

A. a. Family member b. Stranger

c. Friend d. Colleague

e. Can be anyone

f. Others (please specify) __________________________

B. a. Smoker b. Non-smoker c. Don’t know

C. a. Drinker b. Non-drinker c. Don’t know

D. a. Young person (≤ 30 yrs) b. Middle aged (30-50 years)

c. Elderly person (> 50 yrs) d. Don’t know

E. a. Mentally retarded person b. Mentally sound people c. Don’t know

F. a. Physically disabled b. Not physically disabled c. Don’t know

G. a. Person belonging to same religion

b. Person belonging to different religion

c. Don’t know

17. Which of the following factor holds the greatest importance near you when **donating** an organ? (*Choose one option*)

a. Relation to the person b. Age of recipient

c. Religion of recipient d. Health status of recipient

e. Substance abuse of the body
 f. Assurance of the respectful treatment of the organ

g. None of the above

18. For **living donation**, who should give consent?

a. Donor b. His family

c. His spouse d. His friends

e. His doctor f. Others (specify) _________________

19. For **donation after death**, who should give consent?

a. No one b. Family

c. Spouse d. Doctor

e. Friend f. Others (specify) _______________

20. Who should make such decisions about organ donation in case of **unclaimed dead bodies**?

a. Charitable organization

b. Medical colleges / doctors

c. Police

d. A judge

e. No one

21. Can parents / guardians make substitute decision making for **mentally disabled** persons in the regard of **organ donation**?

a. Yes b. No c. Don’t know

22. Should **organ donation** be promoted?

a. Yes b. No c. Don’t know

23. If you answered **No** to **Q 22**, then why not?

a. Fear that organs could be wasted / mistreated

b. Would not want to be cut open or mutilated

c. Religious beliefs

d. Family/parent refusal

e. Harmful for the donor

f. Fear of postoperative pain

g. Can lead to organ trade / violation of rights

h. Other reason (please specify) ________________________________________________

**Part B:**

24. Do you know of anyone who has **donated** an organ?

a. Family member b. Friend c. Colleague d. No one

d. Others (please specify) _______________________________________

25. Have you ever **donated** an organ?

a. Yes b. No

26. If you answered **Yes** to **Q.25**, then proceed further. Otherwise go directly to Part C. Please specify which organ you donated?

_______________________________________________________________________

27. Please specify the reason for donation?

_____________________________________________________________________________

28. Have you experienced any effects that you attribute to **organ donation?**

a. Yes b. No c. Don’t know

29. If your answer to **Q 28** is **Yes**, please specify the effect you experienced?

**Part C:**

30. The term **‘Organ Donation ’** means?

a. the removal of the [tissues](http://en.wikipedia.org/wiki/Biological_tissue) of the [human body](http://en.wikipedia.org/wiki/Human_body) from a cadaver

b. the removal of the [tissues](http://en.wikipedia.org/wiki/Biological_tissue) of the [human body](http://en.wikipedia.org/wiki/Human_body) from a living donor.

c. the removal of the [tissues](http://en.wikipedia.org/wiki/Biological_tissue) of the [human body](http://en.wikipedia.org/wiki/Human_body) for the purpose of transplantation to another person

d. Can include transfer of cell/ova/fetus/sperm

e. All of the above

f. Others (specify) ________________________________________________________

31. You heard about **organ donation** through which of the following sources? *(You can choose more than one option)*

a. Heard from a doctor b. Internet /online resources

c. TV d. Radio

e. Newspaper or magazines f. Friend or colleague

g. Other (specify) ____________________________

32. Why is organ donation done?

a. To save someone’s life

b. Out of compassion/sympathy

c. For money

d. As a ‘responsibility’

e. Others (specify) _____________________________________________________________

33. What organs can be donated? (You can choose more than one option)

a. Kidney b. Blood

c. Heart d. Eyes

e. Liver f. Skin

g. Bone marrow h. Lungs

j. All of above k. None of the above

i. Others (Please specify) ________________________

34. Does **organ donation** involve any risks?

a. Yes b. No c. Don’t know

35. If you answered **Yes** to **Q 34**, then which risk, in your opinion, is the most important in **organ donation**?

a. Infection b. Bodily weakness

c. Anxiety and depression d. Pain

e. Bleeding f. All of the above

g. None of the above h. Others (specify) ______________________

36. Are you aware of any **local or international legislation** with regards to **organ donation**?

a. Local legislation b. International legislation

c. Both of the above d. None of the above

37. Is there any need for having effective laws to govern the process of **organ donation**?

a. Yes b. No c. Don’t know

***THANKYOU FOR YOUR VALUABLE TIME & EFFORT**

***ANY SUGGESTIONS/OPINIONS REGARDING THE QUESTIONNAIRE & ITS IMPROVEMENT ARE MOST WELCOME**
